# Supplementary material for: Reciprocal rescue of Wolfram syndrome by two causative genes
Source: EMBO Rep. 2025 Apr 3;26(9):2459–82. doi: 10.1038/s44319-025-00436-2 (PMC12069674; doi:10.1038/s44319-025-00436-2)
Supplement: Supplementary file 1 — Appendix [file 44319_2025_436_MOESM1_ESM.pdf]

Appendix for

"Reciprocal rescue of Wolfram syndrome by two causative genes"

Table of Contents

Appendix Figure S1	--	3
Appendix Figure S2	--	4
Appendix Figure S3	--	5
Appendix Figure S4	--	6
Appendix Figure S5	--	7
Appendix Figure S6	--	8
Appendix Figure S7	--	9
Appendix Figure S8	--	10
Appendix Figure S9	--	11
Appendix Figure S10	--	12
Appendix Table S1	--	13

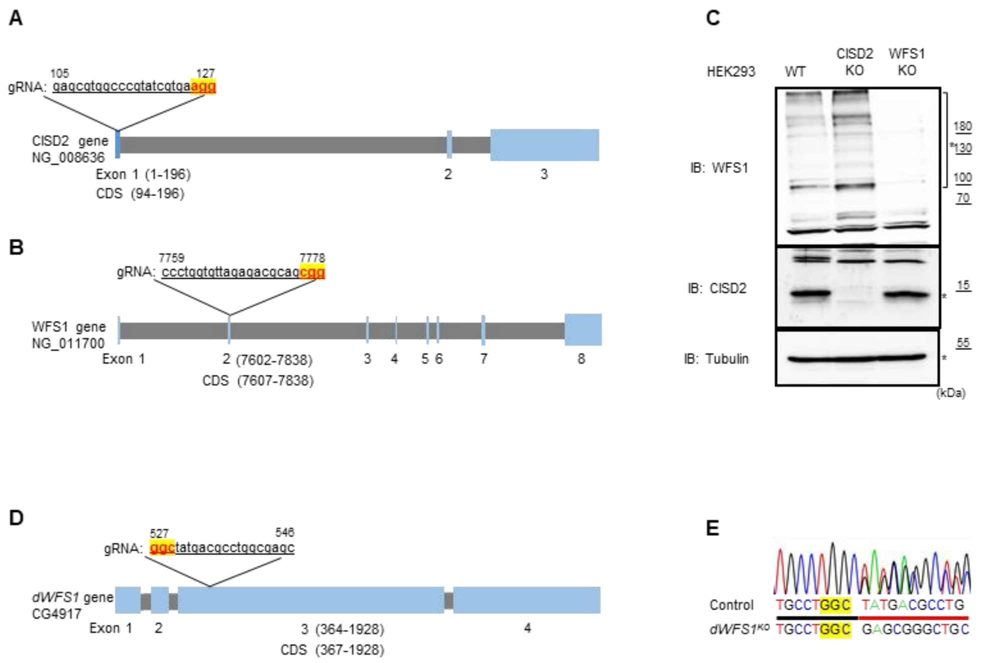

Appendix Figure S1. Generation of WFS1 KO and CISD2 KO HEK293 cells and *dWFS1* mutant *Drosophila* using CRISPR-Cas9 system.

(A) Scheme for generating human WFS1 KO HEK293 cells. (B) Scheme for generating human CISD2 KO HEK293 cells. (A, B) Protospacer adjacent motif (PAM) in the gRNA sequence is indicated in red and highlighted in yellow. (C) Immunoblot analysis of endogenous WFS1 and CISD2 in WT, WFS1 KO, and CISD2 KO HEK293 cells. The asterisks denote the band of interest. (D) Scheme for generating *dWFS1* mutant *Drosophila*, with the gRNA shown and PAM in red and highlighted in yellow. (E) Sequencing analysis to confirm *dWFS1* mutant flies. PAM is highlighted in yellow, and the following double peak sequence indicates a frameshift mutation and successful generation of *dWFS1* knockout mutant.

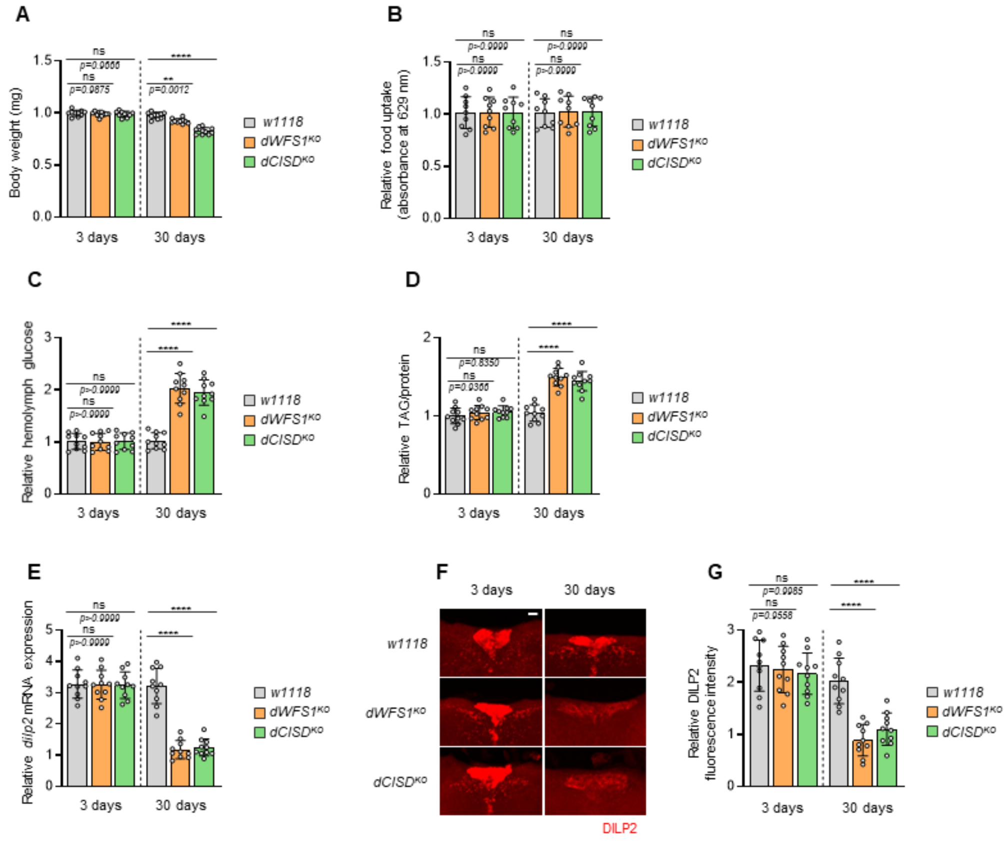

Appendix Figure S2. Loss of *dWFS1* or *dCISD* in *Drosophila* induces phenotypes analogous to type 1 diabetes with age.

(A) Body weight of flies at 3 and 30 days of age (n=10). (B) Feeding assays of 3- and 30-day-old flies starved for 24 hours, then fed a sucrose solution with blue dye for 1 hour. Flies were homogenized and examined for absorbance at 629 nm, normalized to *w1118* control flies (n=10). (C) Relative hemolymph glucose of flies at 3 days and 30 days, normalized to controls (n=10). (D) Relative TAG levels of flies at 3 days and 30 days, normalized to controls (n=10). (E) Relative *dilp2* mRNA expression of flies at 3 and 30 days of age, normalized to *dWFS1* KO flies (n=10). (F) Representative images of 3- and 30-day-old fly brains stained with anti-DILP2. Scale bar, 10 µm (n=10). (G) Quantified fluorescence intensity of (F), normalized to *dCISD* KO flies (n=10). Data information: All figures are representatives of three or more independent experiments. All quantifications were analyzed by one-way ANOVA with Tukey multiple-comparison test. **$p < 0.01$, **** $p < 0.0001$. ns, not significant. All data are presented as mean ± SD.

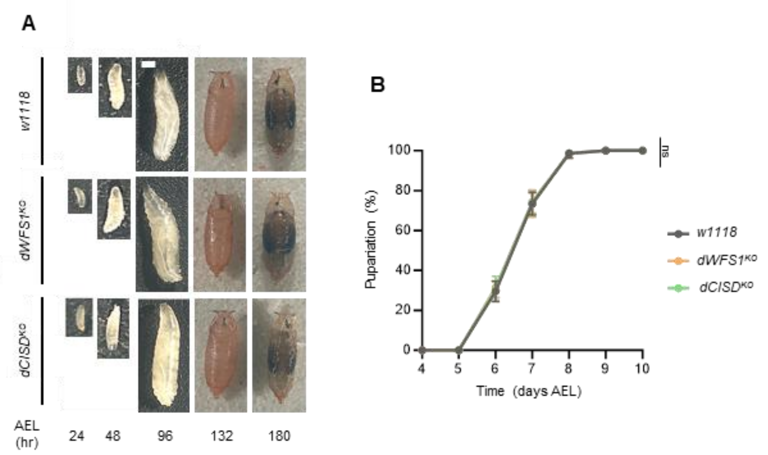

Appendix Figure S3. *Drosophila* lacking *dWFS1* or *dCISD* do not show developmental defects.

(A) Tracking development of *w1118*, *dWFS1* KO, and *dCISD* KO flies at 24, 48, 96, 132, and 180 hours after egg laying (AEL). Scale bar, 0.5 mm. (B) Percentage of pupation of *w1118*, *dWFS1* KO, and *dCISD* KO flies at days AEL (n=100). ns indicates not significant, with p-values of >0.999 (*w1118* vs. *dWFS1* KO) and 0.9998 (*w1118* vs. *dCISD* KO). Data information: The experiment was independently repeated five times. Quantifications were analyzed by one-way ANOVA with Tukey multiple-comparison test. ns, not significant. Data is presented as mean ± SD.

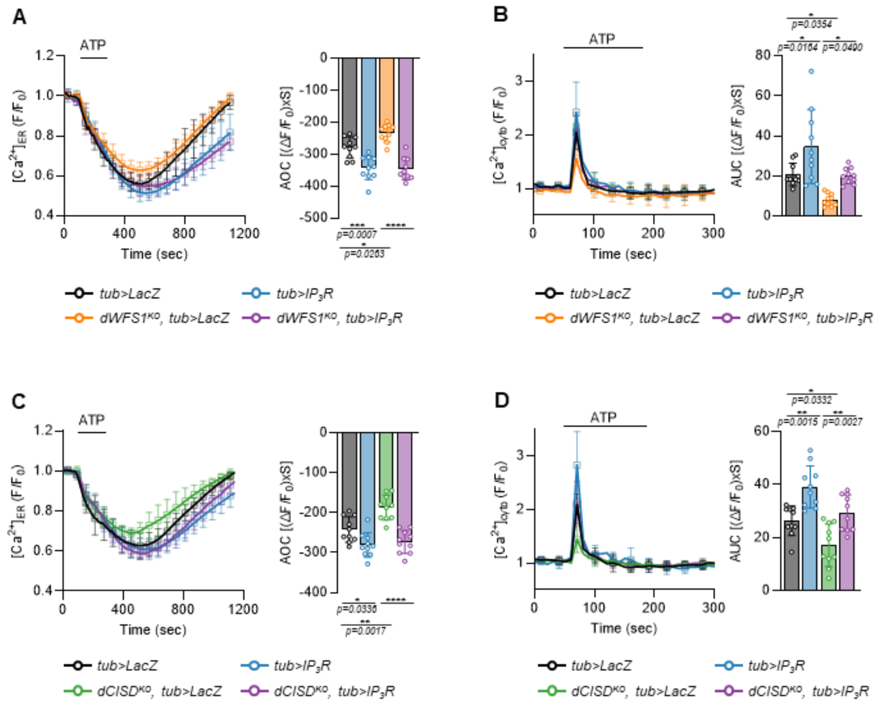

Appendix Figure S4. Overexpression of *IP₃R* restores altered calcium modulation in *dWFS1* and *dCISD* null flies.

(A, B) Measurement of ER calcium (A) and cytosolic calcium (B) modulations in control (*tub>LacZ*, black) and *IP$_3$R* transgenic flies (*tub>IP$_3$R*, blue). Similar experiments were also conducted in *dWFS1* mutant flies (*dWFS1 KO, tub>LacZ*, orange) and *dWFS1* null flies expressing *IP$_3$R* (*dWFS1 KO, tub>IP$_3$R*, purple). (C, D) Measurement of ER calcium (C) and cytosolic calcium (D) modulations in control (*tub>LacZ*, black) and *IP$_3$R* transgenic flies (*tub>IP$_3$R*, blue). Similar experiments were also conducted in *dCISD* mutant flies (*dCISD KO, tub>LacZ*, green) and *dCISD* mutant flies expressing *IP$_3$R* (*dCISD KO, tub>IP$_3$R*, purple). The right-side bar graphs indicate the quantification of the normalized calcium traces using AOC or AUC of calcium fluxes during ATP treatment (n=10). All quantifications were analyzed by one-way ANOVA with Tukey multiple-comparison test. *p < 0.05, **p < 0.01, ***p < 0.001, ****p < 0.0001. All data are presented as mean ± SD.

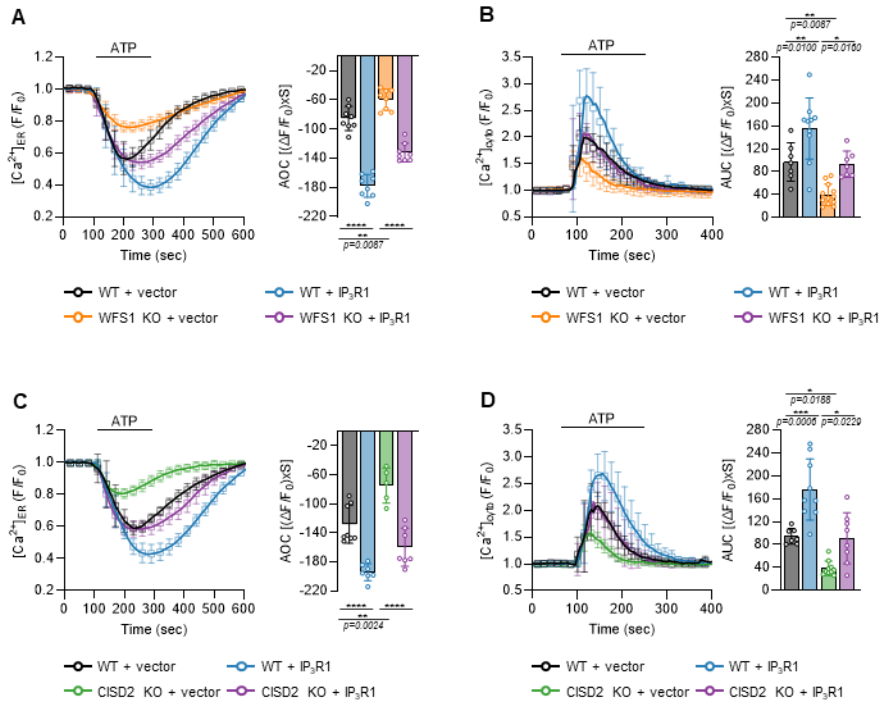

Appendix Figure S5. Overexpression of IP$_3$R1 restores altered calcium modulation in WFS1 and CISD2 lacking cells.

(A) Measurement of ER calcium modulation in WT HEK293 cells transfected with empty vector (black, n=84 cells, 8 coverslips) or *IP$_3$R1* (blue, n=104 cells, 8 coverslips) and WFS1 KO HEK293 cells transfected with empty vector (orange, n=107 cells, 8 coverslips) or *IP$_3$R1* (purple, n=97 cells, 8 coverslips). (B) Measurement of cytosolic calcium modulation in WT HEK293 cells transfected with empty vector (black, n=99 cells, 7 coverslips) or *IP$_3$R1* (blue, n=117 cells, 10 coverslips) and WFS1 KO HEK293 cells transfected with empty vector (orange, n=71 cells, 12 coverslips) or *IP$_3$R1* (purple, n=84 cells, 7 coverslips). (C) Identical experiments measuring ER calcium modulation were conducted in WT HEK293 cells transfected with empty vector (black, n=108 cells, 8 coverslips) or *IP$_3$R1* (blue, n=114 cells, 7 coverslips) and CISD2 KO HEK293 cells transfected with empty vector (green, n=80 cells, 5 coverslips) or *IP$_3$R1* (purple, n=116 cells, 7 coverslips). (D) Identical experiments measuring cytosolic calcium modulation were conducted in WT HEK293 cells transfected with empty vector (black, n=82 cells, 7 coverslips) or *IP$_3$R1* (blue, n=62 cells, 9 coverslips) and CISD2 KO HEK293 cells transfected with empty vector (green, n=54 cells, 10 coverslips) or *IP$_3$R1* (purple, n=61cells, 8 coverslips). The right-side bar graphs indicate the quantification of the normalized calcium traces using AOC or AUC of calcium fluxes during ATP treatment. Data information: All figures are representatives of three or more independent experiments. All quantifications were analyzed by one-way ANOVA with Tukey multiple-comparison test. *$p < 0.05$, **$p < 0.01$, ***$p < 0.001$, ****$p < 0.0001$. All data are presented as mean ± SD.

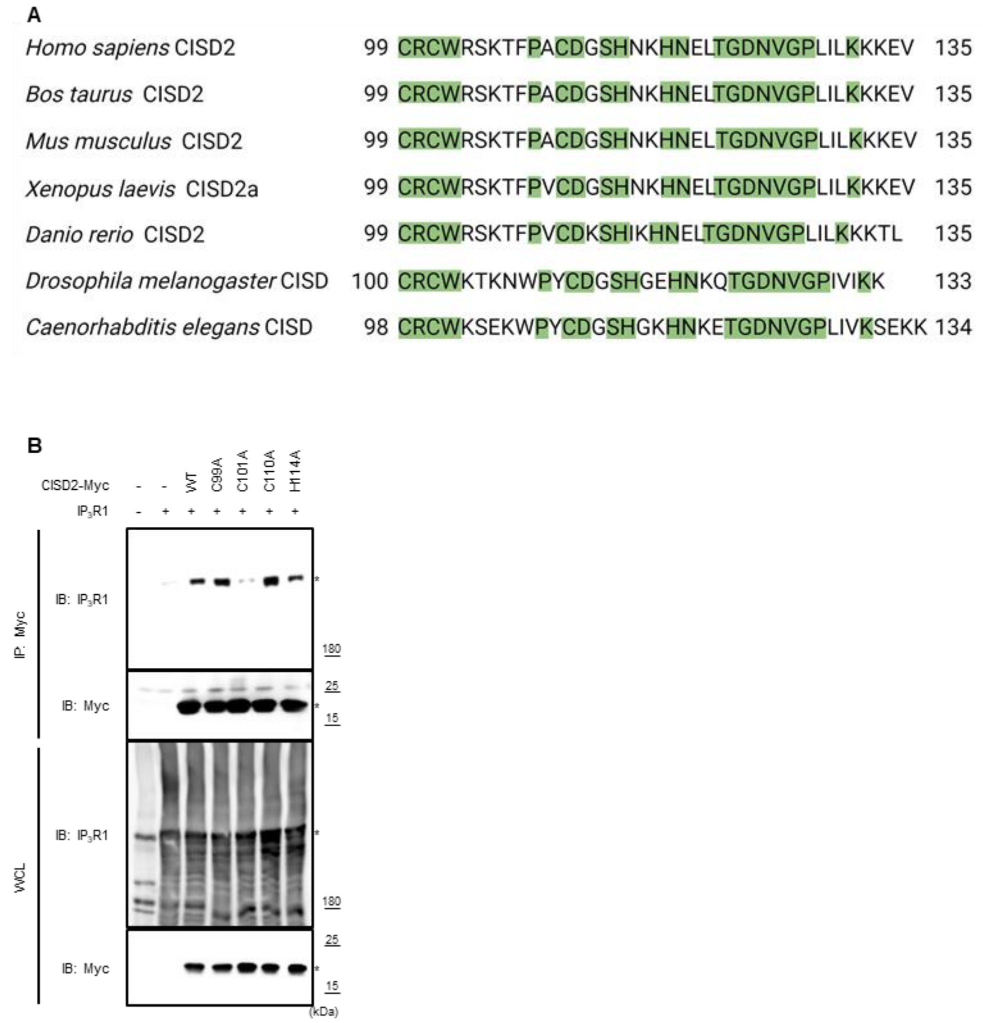

Appendix Figure S6. Among highly conserved Fe-S cluster binding residues in CISD2 across different species, cysteine 101 is essential for the protein-protein interaction with IP_3R1.

(A) Schematic diagram representing protein sequences of CISD2 C-terminus aligned across seven species. Those are *Homo sapiens* (NM_001008388.4), *Bos taurus* (NM_001080338.1), *Mus musculus* (NM_025902.3), *Xenopus laevis* (NM_001089751.1), *Danio rerio* (NM_200383.1), *Drosophila melanogaster* (NM_143427.4), and *Caenorhabditis elegans* (NM_001129176.4). The conserved amino acids are highlighted in green. (B) HEK293T cells were transfected as indicated and cell lysates were subjected to anti-Myc immunoprecipitation followed by immunoblot analysis. The asterisks denote the band of interest.

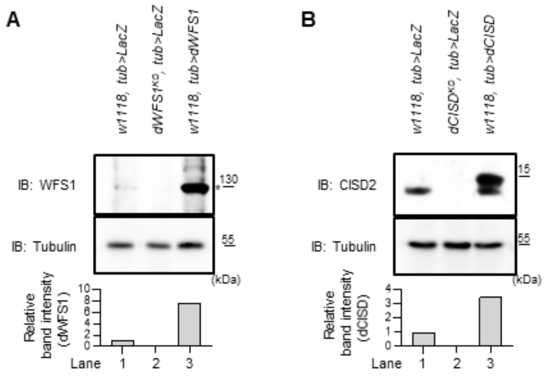

Appendix Figure S7. Protein expression levels in *dWFS1* and *dCISD* transgenic flies.

(A) Whole bodies of *w1118* and *dWFS1* KO flies expressing UAS-*LacZ* or UAS-*dWFS1* were lysed and subjected to immunoblot analysis. (B) Whole bodies of *w1118* and *dCISD* KO flies expressing UAS-*LacZ* or UAS-*dCISD* were lysed and subjected to immunoblot analysis. The upper band indicates Flag-tagged dCISD and the lower band indicates endogenous dCISD. The asterisk denotes the band of interest. Anti-tubulin blot was used as a loading control. Bottom bar graphs represent the band intensity of dWFS1 and dCISD, which were quantified using Image J.

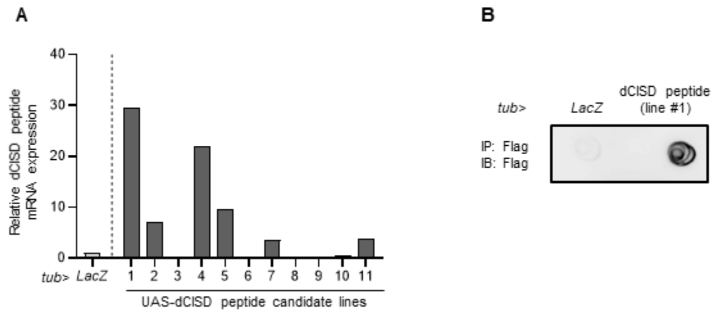

Appendix Figure S8. Generation of dCISD peptide expressing transgenic flies.

(A) Relative mRNA expression of dCISD peptide of candidate UAS-dCISD peptide transgenic lines crossed with *tub-Gal4*, normalized to *rp49*. Candidate line #1 exhibited the highest expression and was thus employed in our experiments. (B) Fly (line #1) whole body lysates were subjected to anti-Flag immunoprecipitation followed by dot blot analysis.

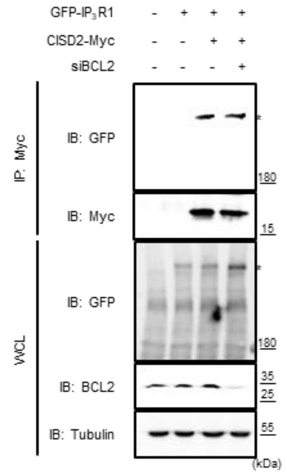

Appendix Figure S9. Knockdown of BCL2 does not affect the interaction between IP$_3$R1 and CISD2.

HEK293T cells were transfected as indicated and cell lysates were subjected to anti-Myc immunoprecipitation followed by immunoblot analysis. The asterisk denotes the band of interest.

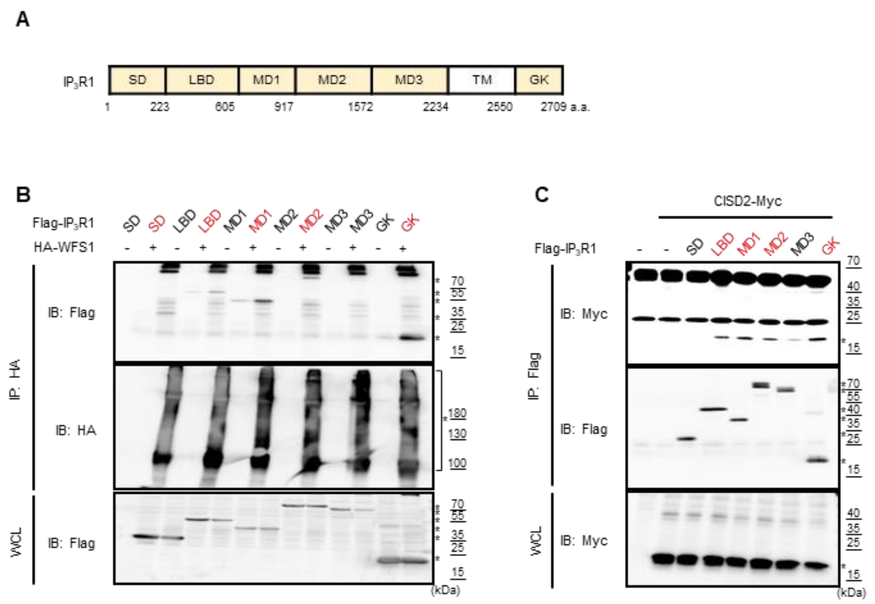

Appendix Figure S10. WFS1 interacts with suppressor domain (SD) domain of IP_3R1 while CISD2 does not.

(A) Schematic diagram of the protein architecture of IP_3R1. The suppressor domain (SD), ligand-binding domain (LBD), modulatory domain (MD) 1, MD2, MD3, transmembrane (TM), and gate-keeping (GK) domains were designated. The truncated forms of IP_3R1 domains that were used for co-IP with WFS1 or CISD2 are indicated in yellow. (B) HEK293 cells were transfected as indicated and cell lysates were subjected to anti-HA immunoprecipitation followed by immunoblot analysis. The truncated mutants of IP_3R1 domains that interacted with WFS1 are highlighted in red. (C) HEK293 cells were transfected as indicated and cell lysates were subjected to anti-Flag immunoprecipitation followed by immunoblot analysis. The truncated mutants of IP_3R1 domains that interacted with CISD2 are highlighted in red. The asterisks denote the band of interest.

Appendix Table S1. Primers used for qPCR analysis.

Primer	Sequence
Drosophila dCISD peptide Forward	5'- GGTCGGAGTACTGTCCTCCG-3'
Drosophila dCISD peptide Reverse	5'- CCTCTAGACTCGCCATGACTG-3'
Drosophila dilp2 Forward	5'-GGGGGTGTACTCAATTCCCT-3'
Drosophila dilp2 Reverse	5'-TGGTTGGTTTGGAACCAGAT-3'
Drosophila rp49 Forward	5'-AGCTTCAAGATGACCATCCG-3'
Drosophila rp49 Reverse	5'-CCAGGAACTTCTTGAATCCG-3'
