# Supplementary figures and images for: Reciprocal rescue of Wolfram syndrome by two causative genes
Source: EMBO Rep. 2025 Apr 3;26(9):2459–82. doi: 10.1038/s44319-025-00436-2 (PMC12069674; doi:10.1038/s44319-025-00436-2)

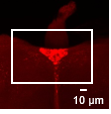

Supplement: Supplementary file 3 — Source data Fig. 1 [file 44319_2025_436_MOESM3_ESM.zip › Figure 1/1H/1H dWFS1 KO, IP3R.png]

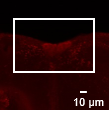

Supplement: Supplementary file 3 — Source data Fig. 1 [file 44319_2025_436_MOESM3_ESM.zip › Figure 1/1H/1H dCISD KO, LacZ.png]

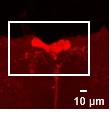

Supplement: Supplementary file 3 — Source data Fig. 1 [file 44319_2025_436_MOESM3_ESM.zip › Figure 1/1H/1H Control, LacZ.png]

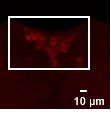

Supplement: Supplementary file 3 — Source data Fig. 1 [file 44319_2025_436_MOESM3_ESM.zip › Figure 1/1H/1H dWFS1 KO, LacZ.png]

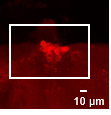

Supplement: Supplementary file 3 — Source data Fig. 1 [file 44319_2025_436_MOESM3_ESM.zip › Figure 1/1H/1H dCISD KO, IP3R.png]

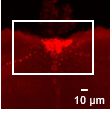

Supplement: Supplementary file 3 — Source data Fig. 1 [file 44319_2025_436_MOESM3_ESM.zip › Figure 1/1H/1H Control, IP3R.png]

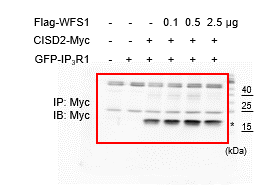

Supplement: Supplementary file 4 — Source data Fig. 2 [file 44319_2025_436_MOESM4_ESM.zip › Figure 2/2G/2G Western IP_Myc.png]

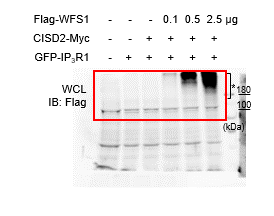

Supplement: Supplementary file 4 — Source data Fig. 2 [file 44319_2025_436_MOESM4_ESM.zip › Figure 2/2G/2G Western WCL_Flag.png]

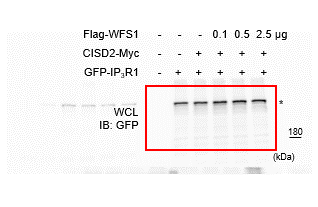

Supplement: Supplementary file 4 — Source data Fig. 2 [file 44319_2025_436_MOESM4_ESM.zip › Figure 2/2G/2G Western WCL_GFP.png]

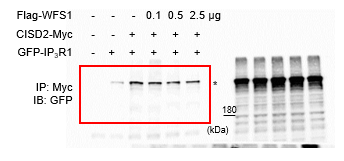

Supplement: Supplementary file 4 — Source data Fig. 2 [file 44319_2025_436_MOESM4_ESM.zip › Figure 2/2G/2G Western IP_GFP.png]

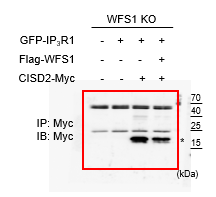

Supplement: Supplementary file 4 — Source data Fig. 2 [file 44319_2025_436_MOESM4_ESM.zip › Figure 2/2I/2D Western IP_Myc.png]

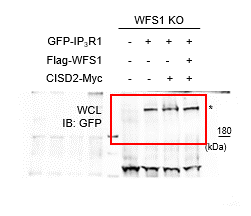

Supplement: Supplementary file 4 — Source data Fig. 2 [file 44319_2025_436_MOESM4_ESM.zip › Figure 2/2I/2D Western WCL_GFP.png]

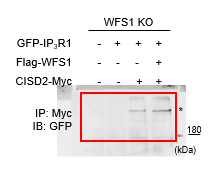

Supplement: Supplementary file 4 — Source data Fig. 2 [file 44319_2025_436_MOESM4_ESM.zip › Figure 2/2I/2D Western IP_GFP.png]

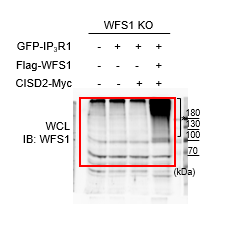

Supplement: Supplementary file 4 — Source data Fig. 2 [file 44319_2025_436_MOESM4_ESM.zip › Figure 2/2I/2D Western WCL_WFS1.png]

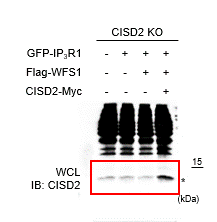

Supplement: Supplementary file 4 — Source data Fig. 2 [file 44319_2025_436_MOESM4_ESM.zip › Figure 2/2H/2D Western WCL_CISD2.png]

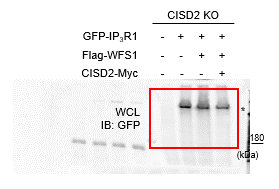

Supplement: Supplementary file 4 — Source data Fig. 2 [file 44319_2025_436_MOESM4_ESM.zip › Figure 2/2H/2D Western WCL_GFP.png]

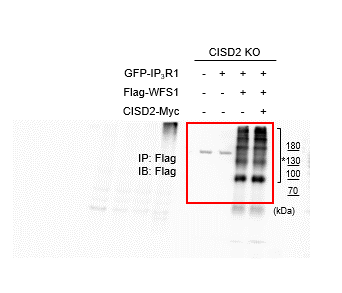

Supplement: Supplementary file 4 — Source data Fig. 2 [file 44319_2025_436_MOESM4_ESM.zip › Figure 2/2H/2D Western IP_Flag.png]

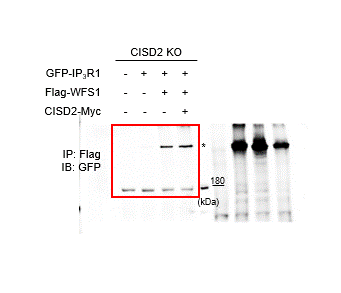

Supplement: Supplementary file 4 — Source data Fig. 2 [file 44319_2025_436_MOESM4_ESM.zip › Figure 2/2H/2D Western IP_GFP.png]

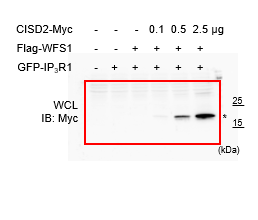

Supplement: Supplementary file 4 — Source data Fig. 2 [file 44319_2025_436_MOESM4_ESM.zip › Figure 2/2F/2F Western WCL_Myc.png]

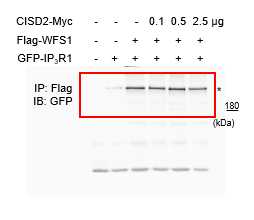

Supplement: Supplementary file 4 — Source data Fig. 2 [file 44319_2025_436_MOESM4_ESM.zip › Figure 2/2F/2F Western IP_GFP.png]

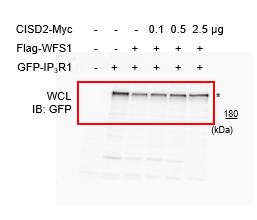

Supplement: Supplementary file 4 — Source data Fig. 2 [file 44319_2025_436_MOESM4_ESM.zip › Figure 2/2F/2F Western WCL_GFP.png]

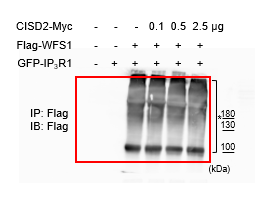

Supplement: Supplementary file 4 — Source data Fig. 2 [file 44319_2025_436_MOESM4_ESM.zip › Figure 2/2F/2F Western IP_Flag.png]

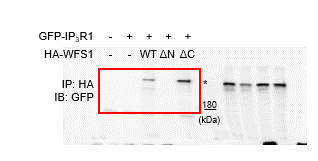

Supplement: Supplementary file 4 — Source data Fig. 2 [file 44319_2025_436_MOESM4_ESM.zip › Figure 2/2C/2C Western IP_GFP.png]

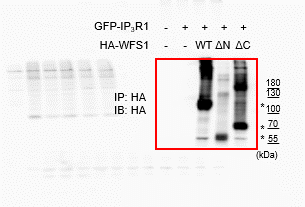

Supplement: Supplementary file 4 — Source data Fig. 2 [file 44319_2025_436_MOESM4_ESM.zip › Figure 2/2C/2C Western IP_HA.png]

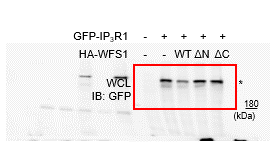

Supplement: Supplementary file 4 — Source data Fig. 2 [file 44319_2025_436_MOESM4_ESM.zip › Figure 2/2C/2C Western WCL_GFP.png]

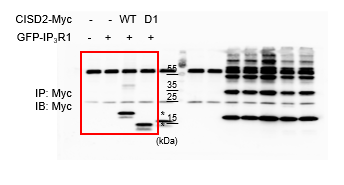

Supplement: Supplementary file 4 — Source data Fig. 2 [file 44319_2025_436_MOESM4_ESM.zip › Figure 2/2D/2D Western IP_Myc.png]

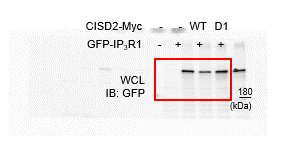

Supplement: Supplementary file 4 — Source data Fig. 2 [file 44319_2025_436_MOESM4_ESM.zip › Figure 2/2D/2D Western WCL_GFP.png]

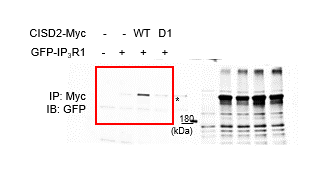

Supplement: Supplementary file 4 — Source data Fig. 2 [file 44319_2025_436_MOESM4_ESM.zip › Figure 2/2D/2D Western IP_GFP.png]

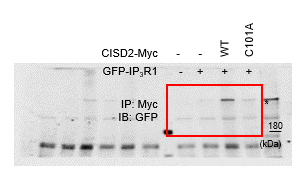

Supplement: Supplementary file 4 — Source data Fig. 2 [file 44319_2025_436_MOESM4_ESM.zip › Figure 2/2E/2E Western IP_GFP.png]

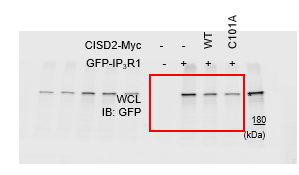

Supplement: Supplementary file 4 — Source data Fig. 2 [file 44319_2025_436_MOESM4_ESM.zip › Figure 2/2E/2E Western WCL_GFP.png]

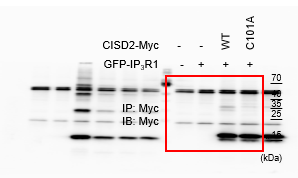

Supplement: Supplementary file 4 — Source data Fig. 2 [file 44319_2025_436_MOESM4_ESM.zip › Figure 2/2E/2E Western IP_Myc.png]

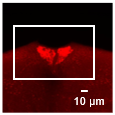

Supplement: Supplementary file 6 — Source data Fig. 4 [file 44319_2025_436_MOESM6_ESM.zip › Figure 4/4I/4I dWFS1 KO, dCISD.png]

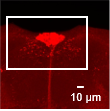

Supplement: Supplementary file 6 — Source data Fig. 4 [file 44319_2025_436_MOESM6_ESM.zip › Figure 4/4I/4I Control, LacZ.png]

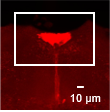

Supplement: Supplementary file 6 — Source data Fig. 4 [file 44319_2025_436_MOESM6_ESM.zip › Figure 4/4I/4I Control, dWFS1.png]

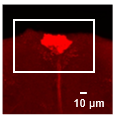

Supplement: Supplementary file 6 — Source data Fig. 4 [file 44319_2025_436_MOESM6_ESM.zip › Figure 4/4I/4I dCISD KO, dCISD.png]

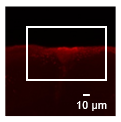

Supplement: Supplementary file 6 — Source data Fig. 4 [file 44319_2025_436_MOESM6_ESM.zip › Figure 4/4I/4I dWFS1 KO, LacZ.png]

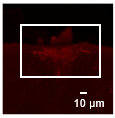

Supplement: Supplementary file 6 — Source data Fig. 4 [file 44319_2025_436_MOESM6_ESM.zip › Figure 4/4I/4I dCISD KO, LacZ.png]

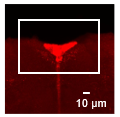

Supplement: Supplementary file 6 — Source data Fig. 4 [file 44319_2025_436_MOESM6_ESM.zip › Figure 4/4I/4I dWFS1 KO, dWFS1.png]

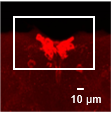

Supplement: Supplementary file 6 — Source data Fig. 4 [file 44319_2025_436_MOESM6_ESM.zip › Figure 4/4I/4I Control, dCISD.png]

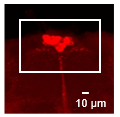

Supplement: Supplementary file 6 — Source data Fig. 4 [file 44319_2025_436_MOESM6_ESM.zip › Figure 4/4I/4I dCISD KO, dWFS1.png]

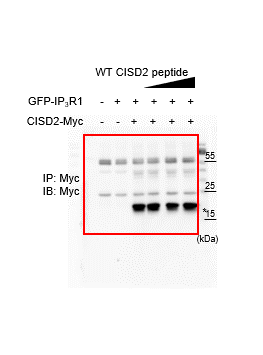

Supplement: Supplementary file 7 — Source data Fig. 5 [file 44319_2025_436_MOESM7_ESM.zip › Figure 5/5B/5B Western_left_IP_Myc.png]

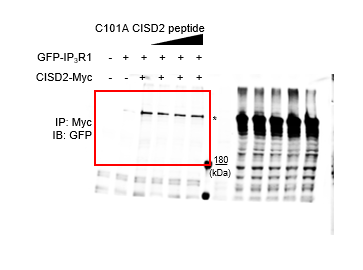

Supplement: Supplementary file 7 — Source data Fig. 5 [file 44319_2025_436_MOESM7_ESM.zip › Figure 5/5B/5B Western_right_IP_GFP.png]

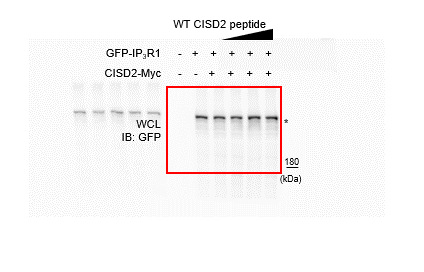

Supplement: Supplementary file 7 — Source data Fig. 5 [file 44319_2025_436_MOESM7_ESM.zip › Figure 5/5B/5B Western_left_WCL_GFP.png]

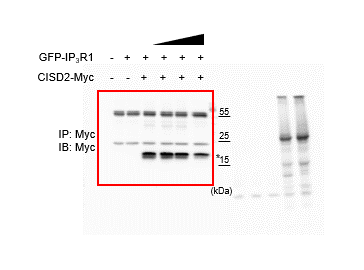

Supplement: Supplementary file 7 — Source data Fig. 5 [file 44319_2025_436_MOESM7_ESM.zip › Figure 5/5B/5B Western_right_IP_Myc.png]

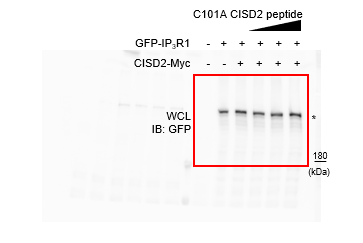

Supplement: Supplementary file 7 — Source data Fig. 5 [file 44319_2025_436_MOESM7_ESM.zip › Figure 5/5B/5B Western_right_WCL_GFP.png]

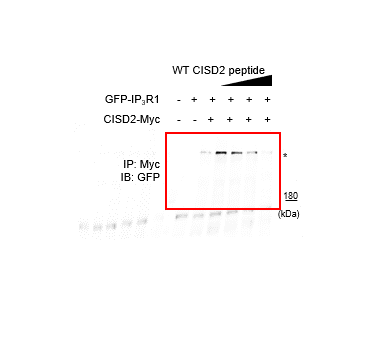

Supplement: Supplementary file 7 — Source data Fig. 5 [file 44319_2025_436_MOESM7_ESM.zip › Figure 5/5B/5B Western_left_IP_GFP.png]

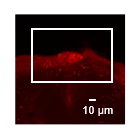

Supplement: Supplementary file 8 — Source data Fig. 6 [file 44319_2025_436_MOESM8_ESM.zip › Figure 6/6F/6F dCISD KO, LacZ.png]

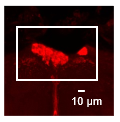

Supplement: Supplementary file 8 — Source data Fig. 6 [file 44319_2025_436_MOESM8_ESM.zip › Figure 6/6F/6F Control, LacZ.png]

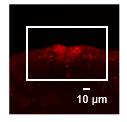

Supplement: Supplementary file 8 — Source data Fig. 6 [file 44319_2025_436_MOESM8_ESM.zip › Figure 6/6F/6F dWFS1 KO, LacZ.png]

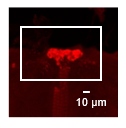

Supplement: Supplementary file 8 — Source data Fig. 6 [file 44319_2025_436_MOESM8_ESM.zip › Figure 6/6F/6F Control, dCISD peptide.png]

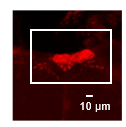

Supplement: Supplementary file 8 — Source data Fig. 6 [file 44319_2025_436_MOESM8_ESM.zip › Figure 6/6F/6F dCISD KO, dCISD peptide.png]

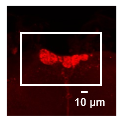

Supplement: Supplementary file 8 — Source data Fig. 6 [file 44319_2025_436_MOESM8_ESM.zip › Figure 6/6F/6F dWFS1 KO, dCISD peptide.png]
