# Supplementary material for: Reciprocal rescue of Wolfram syndrome by two causative genes
Source: EMBO Rep. 2025 Apr 3;26(9):2459–82. doi: 10.1038/s44319-025-00436-2 (PMC12069674; doi:10.1038/s44319-025-00436-2)
Supplement: Supplementary file 9 — Expanded View Figures [file 44319_2025_436_MOESM9_ESM.pdf]

Expanded View Figures

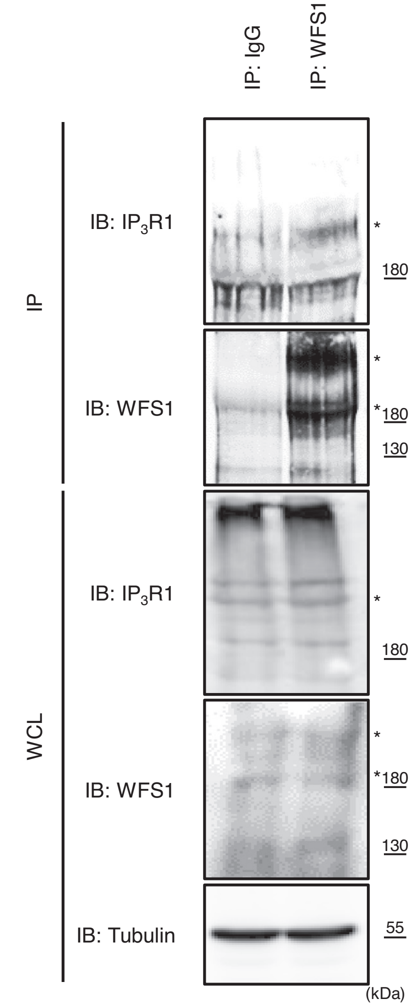

Figure EV1. WFS1 interacts with IP$_3$R1 at the endogenous level.

HEK293 cell lysates were subjected to anti-IgG or anti-WFS1 immunoprecipitation followed by immunoblot analysis. The asterisks denote the band of interest.

 © The Author(s)

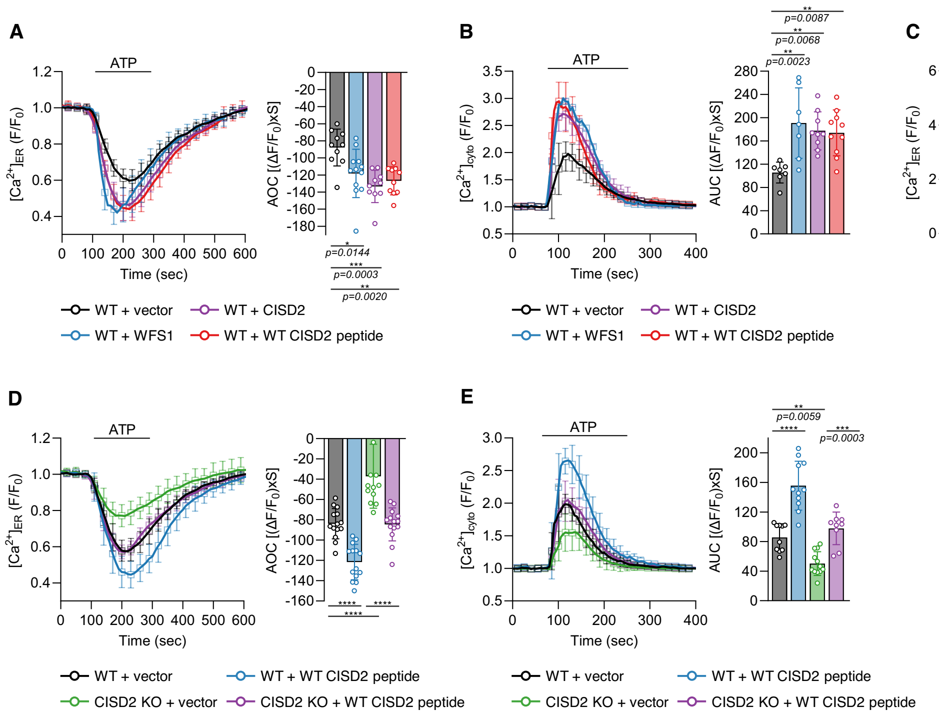

Figure EV2. IP_3R activity is elevated by overexpression of WFS1, CISD2, or CISD2 peptide.

(**A**) Measurement of ER calcium modulation in WT HEK293 cells transfected with empty vector (black, $n = 73$ cells, 10 coverslips), *WFS1* (blue, $n = 54$ cells, 12 coverslips), *CISD2* (purple, $n = 59$ cells, 10 coverslips) or *WT CISD2 peptide* (red, $n = 50$ cells, 10 coverslips). (**B**) Measurement of cytosolic calcium modulation in WT HEK293 cells transfected with empty vector (black, $n = 113$ cells, 7 coverslips), *WFS1* (blue, $n = 105$ cells, 7 coverslips), *CISD2* (purple, $n = 109$ cells, 9 coverslips) or *WT CISD2 peptide* (red, $n = 126$ cells, 10 coverslips). The right-side bar graphs indicate the quantification of the normalized calcium traces using AOC or AUC of calcium fluxes during ATP treatment. (**C**) Measurement of the IP_3R activity of WT HEK293 cells transfected with empty vector (black, $n = 91$ cells, 12 coverslips), *WFS1* (blue, $n = 53$ cells, 12 coverslips), *CISD2* (purple, $n = 72$ cells, 11 coverslips), or *WT CISD2 peptide* (red, $n = 50$ cells, 11 coverslips). The right-side bar graph represents the magnitude of the change during IP_3 treatment. (**D**) Measurement of ER calcium modulation in WT HEK293 cells transfected with empty vector (black, $n = 129$ cells, 14 coverslips) or *WT CISD2 peptide* (blue, $n = 117$ cells, 14 coverslips) and CISD2 KO HEK293 cells transfected with empty vector (green, $n = 146$ cells, 13 coverslips) or *WT CISD2 peptide* (purple, $n = 133$ cells, 14 coverslips). (**E**) Measurement of cytosolic calcium modulation in WT HEK293 cells transfected with empty vector (black, $n = 119$ cells, 10 coverslips) or *WT CISD2 peptide* (blue, $n = 98$ cells, 11 coverslips) and CISD2 KO HEK293 cells transfected with empty vector (green, $n = 113$ cells, 12 coverslips) or *WT CISD2 peptide* (purple, $n = 119$ cells, 9 coverslips). The right-side bar graphs indicate the quantification of the normalized calcium traces using AOC or AUC of calcium fluxes during ATP treatment. Data information: All figures are representatives of three or more independent experiments. All quantifications were analyzed by one-way ANOVA with Tukey multiple-comparison test. *$p < 0.05$, **$p < 0.01$, ***$p < 0.001$, ****$p < 0.0001$. All data are presented as mean ± SD.

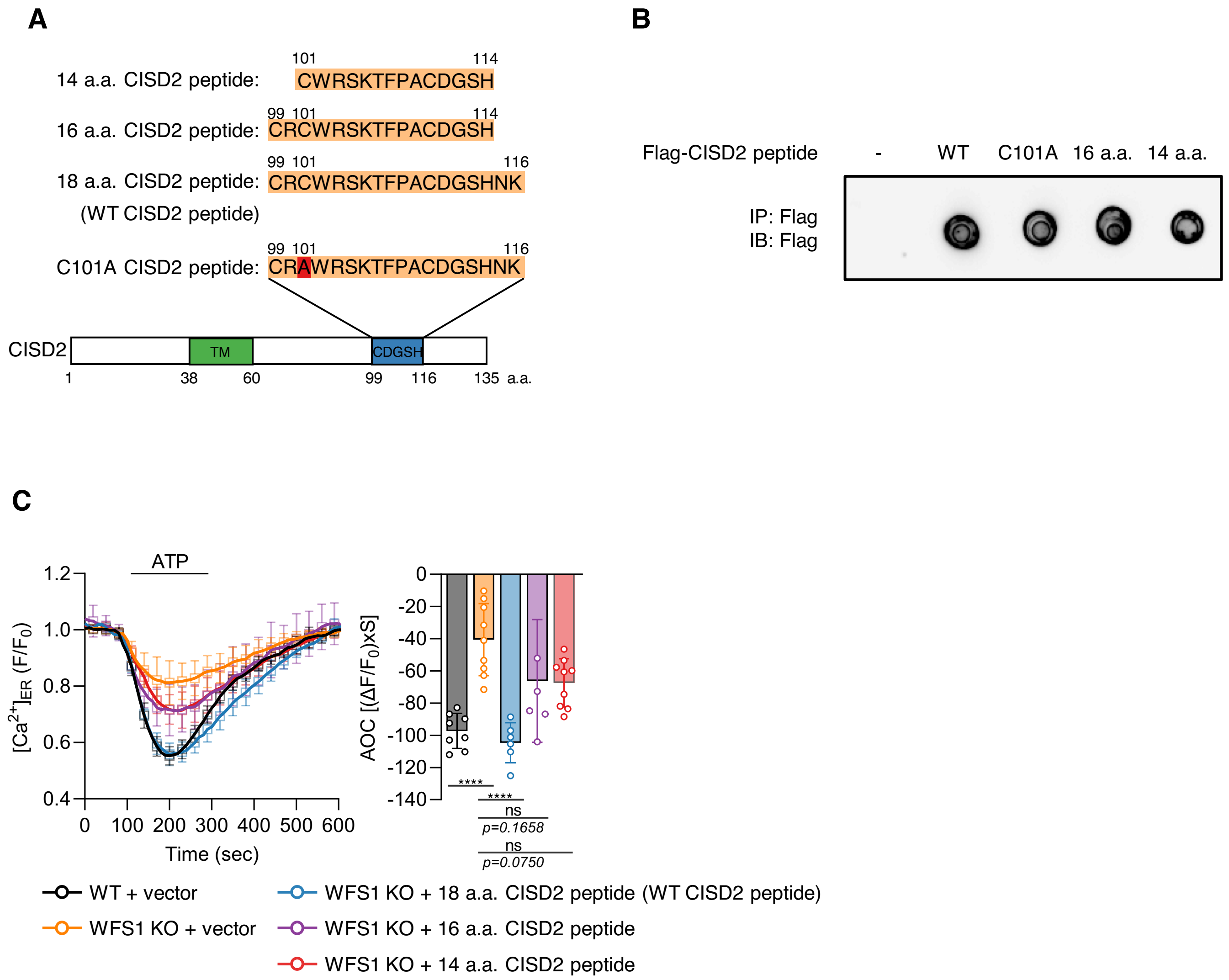

Figure EV3. Overexpression of 18 amino acid CISD2 peptide, but not 16 and 14 amino acid CISD2 peptide, rescues altered calcium modulation in WFS1 knockout cells.

(**A**) Schematic diagram showing various forms of CISD2 peptides. (**B**) HEK293T cells were transfected as indicated and cell lysates were subjected to anti-Flag immunoprecipitation followed by dot blot analysis. (**C**) Measurement of ER calcium release in WT HEK293 cells transfected with empty vector (black, $n = 60$ cells, 8 coverslips) and WFS1 KO HEK293 cells transfected with empty vector (orange, $n = 81$ cells, 9 coverslips), *18 amino acid CISD2 peptide* (referred to as WT CISD2 peptide) (blue, $n = 50$ cells, 6 coverslips), *16 amino acid CISD2 peptide* (purple, $n = 60$ cells, 6 coverslips) or *14 amino acid CISD2 peptide* (red, $n = 72$ cells, 9 coverslips). The right-side bar graphs indicate the quantification of the normalized calcium traces using AOC of calcium fluxes during ATP treatment. Data information: All figures are representatives of three or more independent experiments. All quantifications were analyzed by one-way ANOVA with Tukey multiple-comparison test. ****$p < 0.0001$. ns, not significant. All data are presented as mean ± SD.

 © The Author(s)

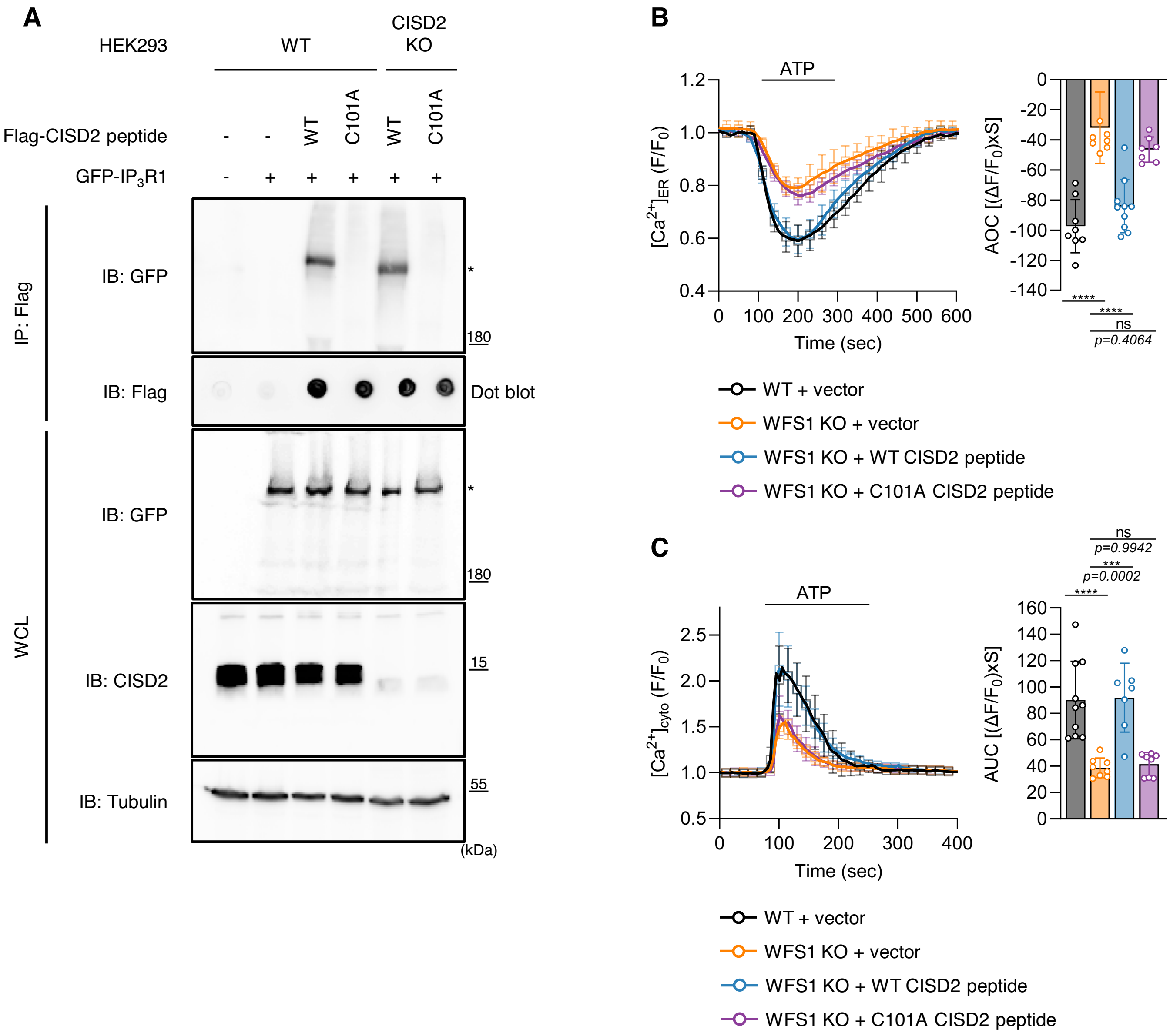

Figure EV4. WT CISD2 peptide, but not C101A CISD2 peptide, directly binds to IP$_3$R1 and rescues altered calcium modulation in WFS1 knockout cells.

(**A**) WT and CISD2 KO HEK293 cells were transfected as indicated and cell lysates were subjected to anti-Flag immunoprecipitation followed by immunoblot analysis or dot blot analysis. (**B**) Measurement of ER calcium modulation in WT HEK293 cells transfected with empty vector (black, $n = 86$ cells, 8 coverslips) and WFS1 KO HEK293 cells transfected with empty vector (orange, $n = 94$ cells, 8 coverslips), *WT CISD2 peptide* (blue, $n = 116$ cells, 10 coverslips), or *C101A CISD2 peptide* (purple, $n = 98$ cells, 7 coverslips). (**C**) Measurement of cytosolic calcium modulation in WT HEK293 cells transfected with empty vector (black, $n = 90$ cells, 10 coverslips) and WFS1 KO HEK293 cells transfected with empty vector (orange, $n = 65$ cells, 8 coverslips), *WT CISD2 peptide* (blue, $n = 96$ cells, 7 coverslips), or *C101A CISD2 peptide* (purple, $n = 91$ cells, 8 coverslips). The right-side bar graphs indicate the quantification of the normalized calcium traces using AOC or AUC calcium fluxes during ATP treatment. Data information: All figures are representatives of three or more independent experiments. All quantifications were analyzed by one-way ANOVA with Tukey multiple-comparison test. ***$p < 0.001$, ****$p < 0.0001$. ns, not significant. All data are presented as mean ± SD.

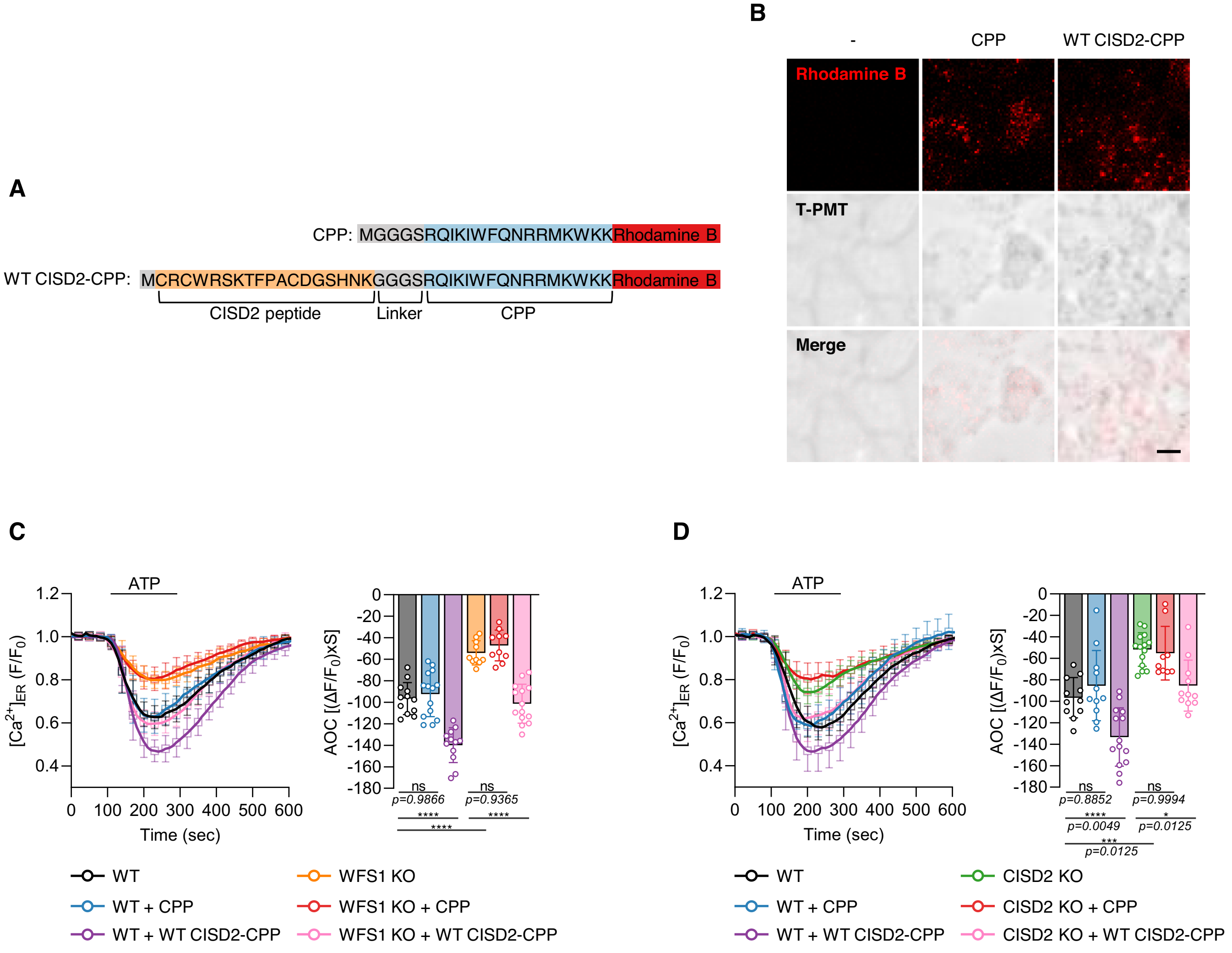

Figure EV5. Treatment of CISD2 cell-penetrating peptide rescues altered calcium modulation in WFS1 or CISD2 knockout cells.

(**A**) Schematic diagram representing cell-penetrating peptide (CPP) and WT CISD2-CPP. (**B**) Representative images of cells incubated with or without cell-penetrating peptides. Peptide uptake was visualized via Rhodamine B fluorescence (red), transmitted-photomultiplier tube (T-PMT) as brightfield, and merged images. Scale bar, 10 µm. (**C**) ER calcium modulation of WT HEK293 cells was measured after incubation with pure buffer (black, $n = 96$ cells, 12 coverslips), 10 µM CPP (blue, $n = 97$ cells, 14 coverslips), or 10 µM WT CISD2-CPP (purple, $n = 92$ cells, 12 coverslips) at 37 °C for 1 h. ER calcium modulation of WFS1 KO cells was measured after incubation with pure buffer (orange, $n = 107$ cells, 10 coverslips), 10 µM CPP (red, $n = 95$ cells, 10 coverslips), or 10 µM WT CISD2-CPP (pink, $n = 112$ cells, 14 coverslips) at 37 °C for 1 h. (**D**) ER calcium modulation of WT HEK293 cells was measured after incubation with pure buffer (black, $n = 107$ cells, 11 coverslips), 10 µM CPP (blue, $n = 85$ cells, 11 coverslips), or 10 µM WT CISD2-CPP (purple, $n = 83$ cells, 14 coverslips) at 37 °C for 1 h. ER calcium modulation of CISD2 KO cells was measured after incubation with pure buffer (green, $n = 84$ cells, 14 coverslips), 10 µM CPP (red, $n = 93$ cells, 9 coverslips), or 10 µM WT CISD2-CPP (pink, $n = 117$ cells, 11 coverslips) at 37 °C for 1 h. The right-side bar graphs indicate the quantification of the normalized calcium traces using AOC of calcium fluxes during ATP treatment. Data information: All figures are representatives of three or more independent experiments. All quantifications were analyzed by one-way ANOVA with Tukey multiple-comparison test. $^{*}p < 0.05$, $^{***}p < 0.001$, $^{****}p < 0.0001$. ns, not significant. All data are presented as mean ± SD.

© The Author(s)
